# TIMP1 is an early biomarker for detection and prognosis of lung cancer

**DOI:** 10.1002/ctm2.1391

**Published:** 2023-09-27

**Authors:** Ezequiel Dantas, Anirudh Murthy, Tanvir Ahmed, Mujmmail Ahmed, Shakti Ramsamooj, Maurice A. Hurd, Tiffany Lam, Murtaza Malbari, Christopher Agrusa, Olivier Elemento, Chen Zhang, Darryl J. Pappin, Timothy E. McGraw, Brendon M. Stiles, Nasser K. Altorki, Marcus D. Goncalves

**Affiliations:** ^1^ Division of Endocrinology Department of Medicine Weill Cornell Medicine New York New York USA; ^2^ Meyer Cancer Center Weill Cornell Medicine New York New York USA; ^3^ Weill Cornell Medical College, Weill Cornell Medicine New York New York USA; ^4^ Division of Thoracic Surgery Weill Cornell Medicine New York New York USA; ^5^ Englander Institute for Precision Medicine Institute for Computational Biomedicine Weill Cornell Medicine New York New York USA; ^6^ Department of Physiology and Biophysics Weill Cornell Medicine New York New York USA; ^7^ Department of Pathology and Laboratory Medicine Weill Cornell Medicine New York New York USA; ^8^ Cold Spring Harbor Laboratory Cold Spring Harbor New York USA; ^9^ Department of Biochemistry Weill Cornell Medicine New York New York USA; ^10^ Department of Cardiothoracic and Vascular Surgery Albert Einstein College of Medicine Bronx New York USA

**Keywords:** biomarkers, lung cancer, prognosis, TIMP1

## Abstract

**Background:**

Lung cancer remains the major cause of cancer‐related deaths worldwide. Early stages of lung cancer are characterized by long asymptomatic periods that are ineffectively identified with the current screening programs. This deficiency represents a lost opportunity to improve the overall survival of patients. Serum biomarkers are among the most effective strategies for cancer screening and follow up.

**Methods:**

Using bead‐based multiplexing assays we screened plasma and tumours of the KrasG12D/+; Lkb1f/f (KL) mouse model of lung cancer for cytokines that could be used as biomarkers. We identified tissue inhibitor of metalloproteinase 1 (TIMP1) as an early biomarker and validated this finding in the plasma of lung cancer patients. We used immunohistochemistry (IHC), previously published single‐cell RNA‐seq and bulk RNA‐seq data to assess the source and expression of TIMP1in the tumour. The prognostic value of TIMP1 was assessed using publicly available human proteomic and transcriptomic databases.

**Results:**

We found that TIMP1 is a tumour‐secreted protein with high sensitivity and specificity for aggressive cancer, even at early stages in mice. We showed that TIMP1 levels in the tumour and serum correlate with tumour burden and worse survival in mice. We validated this finding using clinical samples from our institution and publicly available human proteomic and transcriptomic databases. These data support the finding that high tumour expression of TIMP1 correlates with an unfavorable prognosis in lung cancer patients.

**Conclusion:**

TIMP1 is a suitable biomarker for lung cancer detection.

## Graphical abstract

Lung cancer remains the major cause of cancer‐related deaths worldwide. We found that the tissue inhibitor of metalloproteinase 1 (TIMP1) is a tumour‐secreted protein with high sensitivity and specificity for aggressive cancer, even at early stages in mice. We show that TIMP1 levels in the tumour and serum correlate with tumour burden and worse survival in mice. We validated this finding using clinical samples from our institution and publicly available human proteomic and transcriptomic databases. These data support the finding that high tumour expression of TIMP1 correlates with an unfavorable prognosis in lung cancer patients.

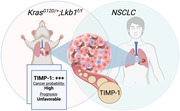

## 1 INTRODUCTION

Lung cancer is the most significant cause of cancer‐related deaths worldwide.^1^ The majority (85%) of lung cancers are classified as non‐small cell lung cancer (NSCLC), with the remaining 15% corresponding to small‐cell lung cancer (SCLC), which has significantly worse prognosis than the latter. The overall 5‐year survival of patients with lung cancer is 20%, but when detected at early stage, survival improves to 60%.^1^, ^2^ Unfortunately, a significant number of patients miss the opportunity to receive curative treatment due to the lack of symptoms during early stages. Early cancer screening programs of the general population using chest radiography or sputum cytology have failed to reduce mortality..^3^, ^4^ Low‐dose computed tomography (LDCT) has helped to identify early stage tumours and improve mortality rates in high risk patients.^2^ Unfortunately, a quarter of the nodules identified by LDCT are benign and this inaccuracy reduces the positive predictive value and limits the therapeutic utility of LDCT in broader populations.^2^ Thus, novel biomarkers are urgently needed to identify and risk‐stratify lung cancers.

The tumour microenvironment is a complex milieu comprised of tumoural, mesenchymal, immune, and stromal cells that constantly interact with the surrounding extracellular matrix (ECM). In fact, cancer cells represent only a fraction of the total tumour mass.^5^ Immune cells represent a dynamic local compartment that plays a decisive role in the outcomes of patients with lung cancer.^6^ The tumour immune microenvironment can assist in the anti‐tumoural response or drive tumour progression by producing cytokines that fuel tumour growth. Moreover, tumour‐secreted cytokines such as IL‐6, IL‐1, IL‐11, LIF can have systemic effects that lead to anorexia, systemic metabolic dysfunction, and cachexia.^7^

The immune system may also influence tumour growth by modulating the interaction of the tumour with the ECM. Tumours with aggressive behaviors are associated with high expression of matrix metalloproteases (MMP), capable of degrading the ECM to facilitate regional invasion and metastasis.^8^ The activity of MMPs is tightly regulated by the secretion of local factors like tissue inhibitors of metalloproteinases (TIMPs). Of the four known TIMP proteins, TIMP1 has been repeatedly associated with cancer progression and poor prognosis in several tumour types like those of the uterus, breast, colon and brain^8^, ^9^ and is also believed to have cytokine‐like activity.^10^

In this study, we hypothesized that tumour‐secreted cytokines and chemokines could serve as biomarkers for lung cancer and predict prognosis. We performed a discovery screen in a well‐established murine model of lung cancer and found that TIMP1 is highly expressed in the tumours of mice, reaching levels high enough in the serum to be used as an early detection biomarker. TIMP1 levels correlated with tumour burden and predicted overall survival in mice and humans with lung cancer.

## 2 MATERIALS AND METHODS

### 2.1 Mice model and tissue collection

The *Kras^G12D/+^; Lkb1^f/f^* mouse model of lung cancer has been previously described.^11^ Mice were housed in a 12‐h light/dark cycle at 22°C room temperature, having free access to rodent chow (PicoLab Rodent 20 5053;Lab Diet) with ad‐libitum drinking water. Tumours were induced in adult (12‐20‐week‐old) male and female mice by intranasal administration of 2.5×10^7^ p.f.u. of Adenovirus CMV‐Cre (Ad5CMV‐Cre) purchased from the University of Iowa Gene Transfer Vector Core (Iowa City, IA). Mice were euthanized when they reached 30% weight loss or developed a poor body composition score (2 or less).

### 2.2 Cytokine measurements

Blood from mice was collected by cardiac puncture and centrifuged at 10 000 × g for 10 min at 4°C; then the serum was stored at −20°C until further analysis. During euthanasia some lungs were dissected to isolate tumours which then were flash frozen in liquid nitrogen and stored at −80°C. To measure cytokines, they were homogenized using a custom‐made lysis buffer (200 mM EDTA, 5 M NaCl, 1%NP‐40, 0,5%Triton X‐100, 10% Glycerol and 1 M Tris‐base). For the initial 44‐ plex screening of cytokines both serum and tumour lysates were sent to Eve Technologies (Calgary, AB). Any other TIMP1 measurements were performed using Luminex kits purchased from R&D.

### 2.3 Histology and immunohistochemistry

Mice lungs were perfused and fixed in 4% paraformaldehyde (PFA) for 24 h and then switched to 70% ethanol prior to paraffin embedding and sectioning at 4 μm. Human tumours were fixed, embedded, and sectioned by the Department of Pathology and Laboratory Medicine at Weill Cornell according to standard of care procedures. Tumours from the KL mice were fixed in 4% PFA overnight and then preserved in 70% ethanol until paraffin embedding, sectioning (4 μm), H&E staining and scanning at Histowiz (Long Island City, NYC, USA). For immunohistochemistry, the slides were dewaxed in Histoclear (National Diagnostics), re‐hydrated and exposed to antigen retrieval using citrate buffer (10 mM Citrate, 0.05% Tween 20, pH 6.0) in a pressure cooker for 10 min. The endogenous peroxidase activity was quenched using 3% hydrogen peroxide in PBS for 5 min. Sections were then blocked using 5% rabbit serum. Slides were incubated for 2 h at room temperature with an anti‐TIMP1 antibody (AF970 for human, and AF980 for mice, R&D, 1:200 dilution). Biotinylated secondary antibody, avidin/biotin reagents (PK‐4100) and DAB peroxidase substrate (SK‐4100) were purchased from Vector Laboratories. Slides were imaged either using an Aperio AT2 (Leica Biosystems) at Histowiz or a Zeiss Axioscope Imager. Tumour area in the lungs of the KL mice was automatically quantified using scanned whole H&E slides and QuPath (0.4.2).^12^

### 2.4 Patient samples

Plasma samples (K2 EDTA) and FFPE tissue blocks from lung cancer patients were obtained from New York Presbyterian Hospital/Weill Cornell Medical College in accordance with a protocol approved by the Institutional Review Board (IRB) (IRB#19‐11021135). Lung adenocarcinoma and adjacent normal tissue used for qPCR analysis were obtained from New York Presbyterian Hospital/Weill Cornell Medical College in accordance with IRB approved protocol number 1008011221. Plasma samples (K2‐EDTA) from healthy donors were obtained from Innovative Research (https://www.innov‐research.com/).

### 2.5 Real‐time PCR

RNA extraction from tumour and healthy margins was done using TRIzol (Thermo Fisher) followed up by an additional clean‐up step using RNeasy kit (Qiagen). cDNA was synthesized using SuperScript VILO Master Mix (Thermo Fisher). TIMP1 qPCR was done using Applied Biosystems SYBR Select Master Mix (Thermo Fisher) and previously published primers (5′−3′ AGCGCCCAGAGAGACACC and CCACTCCGGGCAGGATT).^13^ Relative mRNA expression levels were calculated using the 2^‐ΔΔCt method and normalized to the expression of A549 cells with GAPDH (5′‐ 3 CTTCAACAGCGACACCCACTC C and GTCCACCACCCTGTT GCTGTAG) as housekeeping gene.

### 2.6 Software and statistical analysis

All data were analyzed using RStudio (2023.03.0+386) running R (version 4.0.5) unless stated otherwise. Paired *t*‐test of qPCR data in Figure 3C was done and plotted in GraphPad Prism 9. Kaplan–Meier survival curves were compared using the Log‐Rank test included in the R packages ‘survival’ and ‘surviminer’ for all mouse data and panel C of Figure 4. The R package ‘survminer’ uses the package ‘maxstat’ for calculating the cut‐off point for dichotomization of continuous data using maximally selected rank statistics.^14^ Kaplan‐Meier survival curves for the pan‐cancer combined caBIG, GEO and TCGA databases were plotted and analyzed using the default parameters in (https://kmplot.com/analysis/).^15^ ROC curves and AUC were calculated using the R package plotROC. Threshold, sensitivity and specificity were calculated in the R package pROC, using the method ‘closest.topleft’, assigning a weight of 10 to prioritize sensitivity. Oncoprint of the CPTAC cohort of LUAD^16^ patients was done in cBioPortal (https://www.cbioportal.org/). Epidemiological data in Tables 2, 3, 4 were analyzed using Kruskal–Wallis one‐way analysis of variance followed by Dunn‐Bonferroni's test for multiple comparisons after samples proved not to follow a normal distribution using the Shapiro‐Wilk normality test *p < 0.05, **p < 0.01, ***p < 0.001 and ****p < 0.0001. Graphical abstract was created using Biorender.com.

### 2.7 Bulk RNA‐sequencing

The bulk RNA‐sequencing of the *Kras* ^G12D/+^; *Lkb1* ^f/f^ tumours was previously published and is available at the GEO Database (GSE107470).^17^ Briefly, STAR (v2.4.1d, 2‐pass mode) was used to align reads to the mouse genome (GRCm38), and counts were obtained with HTSeq (v0.6.1). Raw counts were processed using the R package DESeq2 (v.1.30.1). TIMP1 transcriptional correlation analysis was performed with the R package FeatureCorr (v.0.99.0). Volcano plots were done with the R package EnhancedVolcano (v. 1.16.0).

### 2.8 Single‐Cell RNA‐sequencing

Imputed and library‐size normalized expression data of eight primary tumour samples were segmented from data provided in Laughney et. al.^18^ MAGIC imputation was applied as previously described.^18^ Barnes‐hut t‐SNE was calculated using the first twenty principal components (PCs) of the imputed matrix.^19^ t‐SNE coordinates were mapped to cell annotations from the original data. Ridge plots were graphed using the imputed expression of TIMP1. Data segmentation, imputation and t‐SNE were performed using Python 3.9.1. Single‐Cell RNA‐sequencing of lung cancer fibroblasts was published by Hanley et al.^20^ The Seurat object containing scRNA‐sequencing data for lung fibroblasts from this paper is available at https://zenodo.org/record/7400873#.ZGO1RuyZP0o. All graphs were created using RStudio (2023.03.0+386) running R (version 4.0.5).

## 3 RESULTS

### 3.1 TIMP1 is a biomarker of tumour burden in mice with lung cancer

**FIGURE 1:**
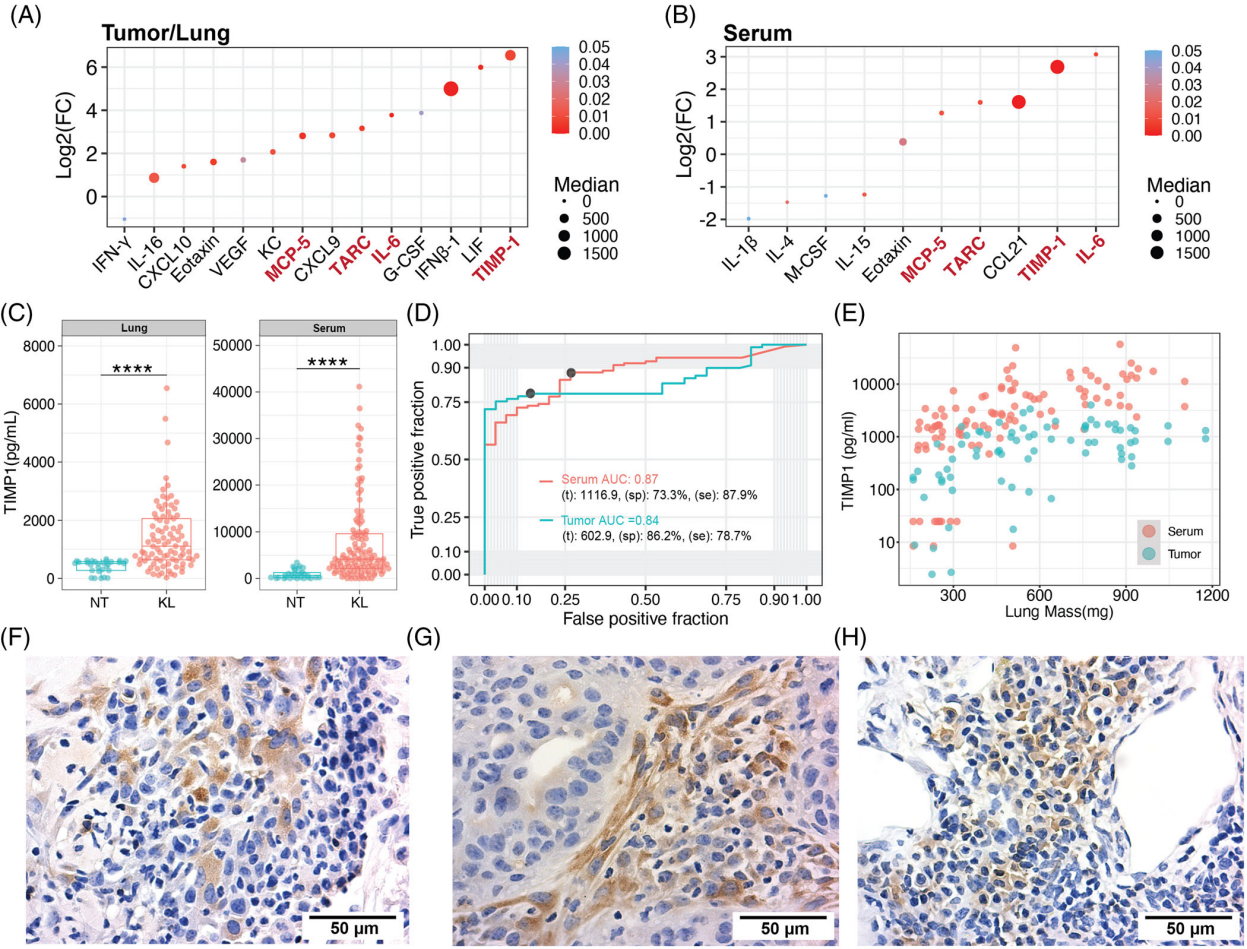
TIMP1 is a biomarker of lung cancer in the KL mice. (A and B) Dot‐Blot of the cytokines that were significantly different when compared to non‐induced mice (WT) in the tumour/lung (A) or serum (B), fold change (FC) is cancer/WT. (C) Concentration levels of TIMP1 in the tumour (*n* = 89) or normal lungs (*n* = 29, left) and serum (WT, *n* = 30, KL, *n* = 127, right) of a larger cohort of KL mice by Luminex. (D) ROC curve of TIMP1 in the serum and tumour of KL mice used in panel (C). Circles show chosen thresholds (t) of TIMP1(pg/mL) used to calculate specificity(sp) and sensitivity(se). (E) Correlation between total lung mass and TIMP1 concentration in the tumour and serum of the KL mice. (F–H) Representative immunohistochemistry (IHC) for TIMP1 expression in the tumours of the KL mice. Comparisons in (A), (B) and (C) were made using Wilcoxon test (*p ≤ 0.05).

We performed a screen for tumour‐secreted cytokines and chemokines using mice carrying inducible *Kras^G12D/+^* and *Stk11/Lkb1^flox/flox^* (KL) alleles, a well‐established genetically engineered mouse model that develops aggressive lung cancer.^11^, ^21^ Following tumour induction using an inhaled adenovirus carrying Cre‐recombinase, blood and tumour tissue were collected following euthanasia. We quantified 45 cytokines and chemokines from the tumour lysates and serum using a Luminex‐based panel (Figure S1A). These levels were compared to those from isogenic control mice that were not induced with Cre (WT). From those that were statistically different, only TARC, MCP‐5, interleukin (IL)‐6, and TIMP1 were significantly higher in both the tumour and serum of the tumour‐bearing mice compared to WT (Figure 1A‐B, and Table 1).

**TABLE 1:** Comparison of cytokines concentration in plasma and tumour of mice with and without tumours.

	Tumour		Serum	
Cytokines	*p*‐value	Log (fold change)	*p*‐value	Log (fold change)
Eotaxin	0.00559	1.606274891	0.0258	0.382896112
G‐CSF	0.0393	3.877885795	0.15	1.470793571
GM‐CSF	0.315	0.876351497	0.537	−0.651112179
IFN‐γ	0.0459	−1.045381952	0.567	−0.281736597
IL.1α	0.667	0.13935876	0.398	1.754692595
IL.1 β	0.953	−0.126785878	0.049	−1.978571638
IL.2	0.486	−0.19251727	0.291	−2.073741186
IL.3	0.755	−0.701918647	0.793	0.865982652
IL.4	0.522	−0.272079545	0.0194	−1.472370674
IL.5	0.778	−0.839535328	0.868	−2.675923439
IL.6	0.00144	3.77740652	0.00759	3.072420063
IL.7	0.127	0.980513782	0.169	−0.504508424
IL.9	0.604	−0.052813716	0.0509	−1.318929505
IL.10	0.266	−0.601281996	0.978	0.013696575
IL.12.p40	0.0659	−0.989972444	0.223	−0.33939932
IL.12.p70	0.327	−0.46062013	0.0653	0.070117309
IL.13	0.496	−0.090981876	0.488	−1.221434468
IL.15	0.614	−0.253310972	0.0137	−1.237514987
IL.17	0.0787	−0.224040274	0.368	−0.347313413
CXCL10	0.00872	1.404893954	0.256	0.479783619
CXCL1	0.0104	2.071131264	0.688	0.451497268
LIF	0.0016	5.99414388	0.0648	4.632621561
CXCL5	0.144	−0.306761647	0.406	−0.47536996
MCP.1	0.906	0.211924388	0.374	−0.003282842
M.CSF	0.406	−0.260927023	0.0483	−1.277568481
MIG	0.00799	2.839536796	0.369	0.269577252
MIP.1α	0.192	−0.292225046	0.279	−1.459328001
MIP.1 β	0.459	−0.253651654	0.233	−1.056040243
MIP.2	0.202	0.761116535	0.0508	−1.097229966
RANTES	0.293	−0.178644632	0.923	0.042778868
TNF‐α	0.532	0.691632852	0.239	−0.217211686
VEGF	0.0317	1.70052426	0.166	1.052300555
EPO	0.369	3.712825527	0.171	1.141821274
CCL21	0.159	0.054906413	4.54E‐05	1.609081177
Fractalkine	0.859	0.386404708	0.3	0.390770115
IFNβ−1	0.000952	4.998235447	0.489	0.881953163
IL.11	0.159	1.895142211	0.0699	1.969375732
IL.16	0.0123	0.871043146	0.0651	1.023630128
IL.20	0.797	0.619868116	0.326	−0.117175075
CCL22	0.646	0.166678595	1	0.034859296
MCP.5	0.00386	2.814263861	0.0112	1.269520154
CCL20	0.847	−0.035576846	0.0838	0.657230886
MIP.3β	0.159	1.395448066	0.143	0.759136741
TARC	0.00559	3.162504669	0.0103	1.596407798
TIMP1	0.00798	6.551911863	0.000113	2.687125921

We performed an operator receiving characteristic (ROC) analysis of these cytokines in the serum to assess their performance as biomarkers and found that TIMP1 had an area under the curve (AUC) of 0.90, the highest of all the analyzed (Figure S1B and C). Next, we expanded our analysis of TIMP1 to a larger cohort of mice (*N* = 127) and confirmed that it continued to be specifically elevated in tumour‐bearing mice (Figure 1C) and performed well as a biomarker (Figure 1D). The serum and tumour levels of TIMP1 correlated with total lung mass (Figure 1E), a good surrogate for tumour burden (Figure S1D), suggesting a direct link between tumour progression and TIMP1 levels. We confirmed that TIMP1 is being produced by the tumour microenvironment by performing immunohistochemistry (IHC) on fixed, paraffin‐embedded lung sections from the tumour‐bearing mice (Figure 1F, G and H). TIMP1 is mainly detected in the tumour‐associated desmoplastic stroma, including spindle‐shaped fibroblasts and macrophages. Additionally, some staining is evidenced in endothelial cells and in poorly‐differentiated, high‐grade tumour cells. These data suggest that TIMP1 is a tumour‐secreted factor that can be used to identify mice with lung cancer.

### 3.2 High TIMP1 levels correlate with poor survival in mice with lung cancer

**FIGURE 2:**
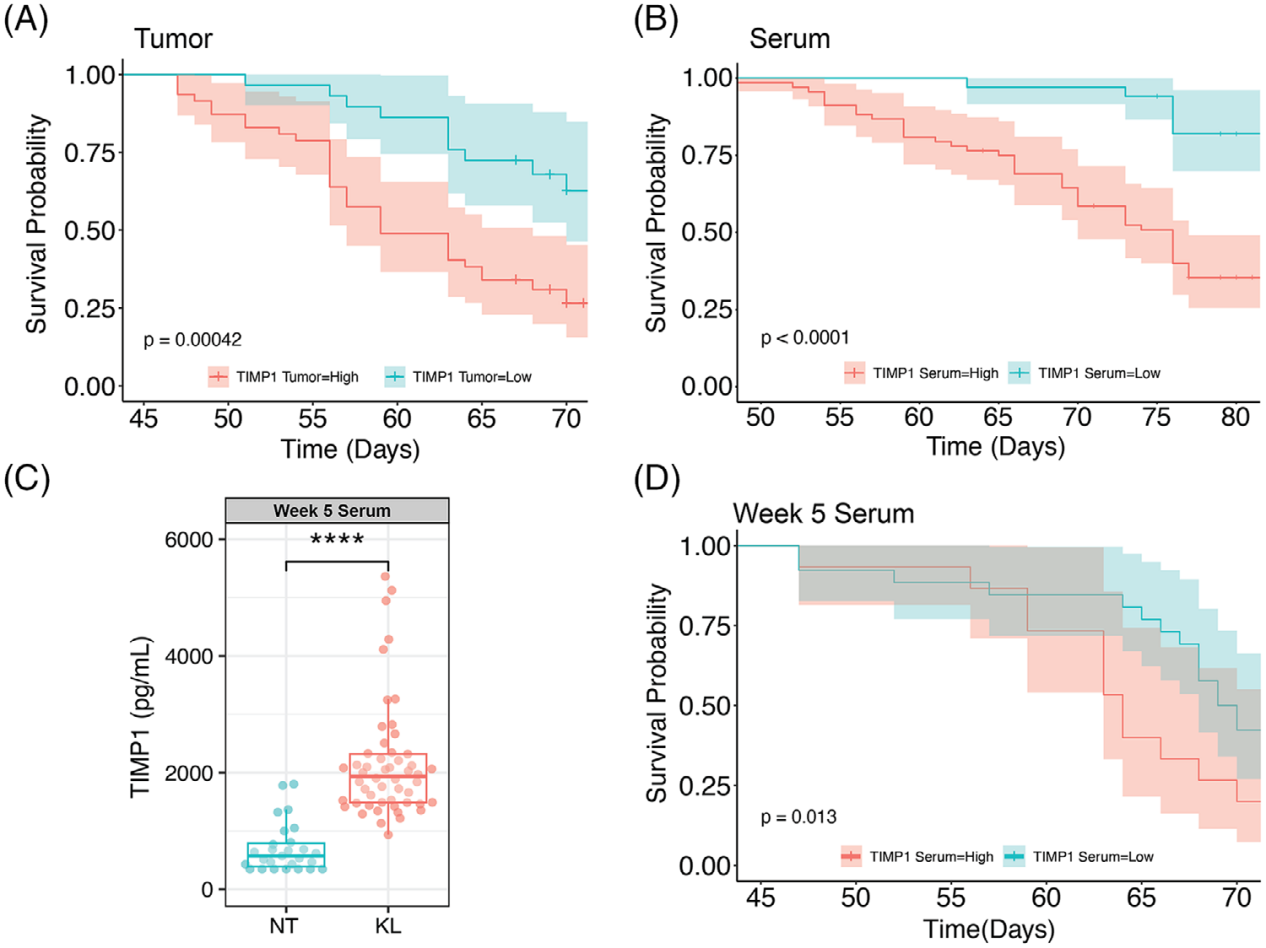
TIMP1 levels in serum and tumour correlate with unfavorable survival probability in mice. (A and B) Kaplan–Meier survival probability graphs using TIMP1 levels in the serum (*n* = 85) (A) and tumour (*n* = 32) (B) as a variable factor. (C) TIMP1 levels in the serum of NT, (*n* = 27) and KL (*n* = 52) mice at week 5 post tumour induction. (D) Kaplan–Meier survival probability graph using TIMP1 levels at week 5 as a variable factor (*n* = 41). Comparison in C was made using Wilcoxon test (*p ≤ 0.05). Comparisons for the KM plots in (A), (B) and (D) were done using the Log‐rank Mantel‐Cox test (*p ≤ 0.05).

Next, we sought to determine if TIMP1 levels could be used to predict overall survival in mice. We measured TIMP1 in serum and tumour lysates from KL mice and performed a Kaplan‐Meyer (KM) survival analysis. The mice with higher TIMP1 levels had worse overall survival rates whether it be in the tumour (Figure 2A) or serum (Figure 2B). These results suggest that TIMP1 could be used as a prognostic biomarker in the KL mice. Given the clinical need for early biomarkers, we measured TIMP1 levels in the serum 5 weeks after tumour induction, a timepoint where the tumour burden is low. At this time, TIMP1 was already significantly increased in the KL mice (Figure 2C) compared to non‐tumour bearing mice (NT), suggesting that TIMP1 is an early marker of tumour development. Also, a KM analysis showed that higher TIMP1 serum concentrations at this early time point correlated with poor survival probability. This data suggests that TIMP1 is an early biomarker and prognostic factor for lung cancer in mice.

### 3.3 TIMP1 is produced by human lung tumours and can be used as a biomarker in humans

**FIGURE 3:**
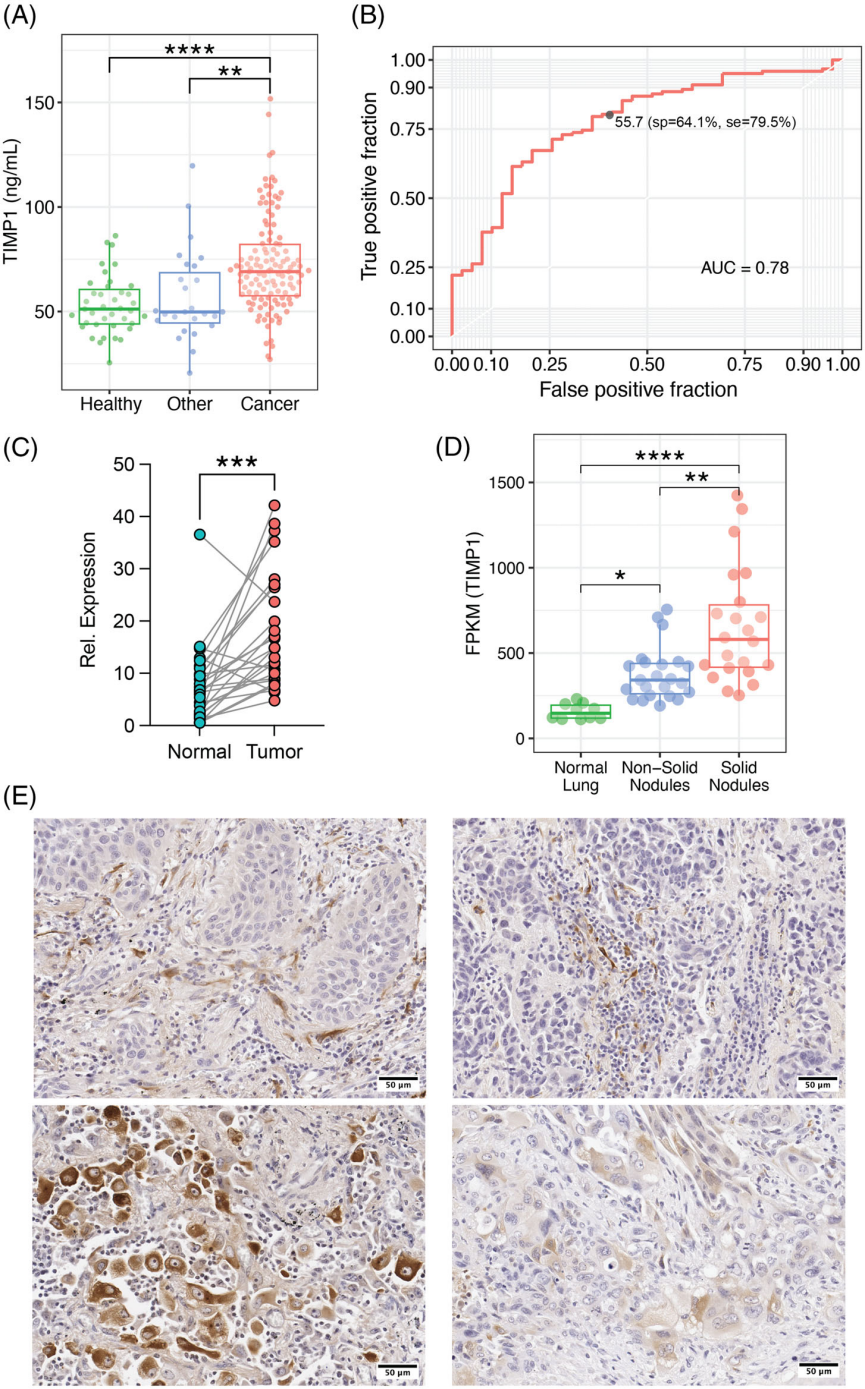
TIMP1 is a biomarker for lung cancer in humans. (A) Concentration of TIMP1 in the plasma of healthy controls (*n* = 39), patients with ‘other’ non‐oncologic thoracic diseases (*n* = 26), and lung cancer patients (*n* = 122). (B) ROC curve using TIMP1 concentration in the plasma of healthy controls and lung cancer patients of panel (A). Circle shows chosen threshold (t) of TIMP1(ng/mL) in plasma used to calculate specificity(sp) and sensitivity(se). (C) Relative expression of TIMP1 mRNA by qPCR in healthy margins (*n* = 26) and tumours (*n* = 26) from lung cancer patients. (D). TIMP1 expression (FPKM) in normal lung (*n* = 10), non‐solid nodules (*n* = 23) and solid nodules using RNA‐seq (*n* = 22). (E) Representative microphotographs of the expression of TIMP1 by immunohistochemistry (IHC) in human lung cancer. Comparisons in (A) and (D) were done using Kruskal–Wallis one‐way analysis of variance followed by Dunn‐Bonferroni's test for multiple comparisons after samples proved not to follow a normal distribution using the Shapiro‐Wilk normality test (*p ≤ 0.05). Comparison in (C) was made using Wilcoxon test (*p ≤ 0.05).

To validate the results found in the KL mice, we measured the concentration of TIMP1 in the plasma of healthy donors, patients with other non‐malignant thoracic pathologies, and patients with lung cancer (Table 2, 3, 4). Similar to what we observed in the mice, TIMP1 was significantly increased in individuals with lung cancer compared to the healthy control group or subjects with other non‐malignant thoracic pathologies (Figure 3A). Furthermore, it also performed well (AUC 0.78) as a biomarker based on a ROC analysis between healthy controls and lung cancer patients (Figure 3B). Although TIMP1 levels did not correlate with histological subtypes, there is a non‐significant statistical trend (*p*‐value = 0.07) for squamous cell carcinomas to have higher levels of TIMP1 (Table 2 and Supp. Figure 2A) and perform slightly better in the ROC analysis when compared to adenocarcinoma (Figure S2B). TIMP1 did not correlate with age, sex or cancer stage (Table 2 and Figure S2C‐D) and its expression at the protein and mRNA levels in the LUAD cohort of the CPTAC^16^ does not correlate with any mutation profile either (Figure S2E). Thus, we conclude that TIMP1 is an agnostic biomarker for lung cancer.

**TABLE 2:** Lung cancer patients.

Features (discrete)	Total	Mean (SD) ng/mL	*p*‐value
Gender*			0.81
Male	52	77.1 (34.2)	
Female	70	71.4 (21.3)	
Clinical Stage			0.78
IA	36	66.3 (19.9)	
IB	24	77.8 (24.0)	
IIA	21	71.4 (20.0)	
IIB	13	75.3 (31.8)	
IIIA	25	82.3 (41.0)	
IV	1	77.6	
0	1	71.8	
TNM			
T			0.71
T1A	37	69.6 (19.7)	
T1B	11	63.6 (21.0)	
T2A	42	79.7 (34.7)	
T2B	8	72.5 (20.0)	
T3	21	74.4 (29.6)	
T4	1	102.0	
N			0.59
N0	78	71.1 (23.2)	
N1	24	73.1 (22.4)	
N2	19	86.1 (44.5)	
M			0.69
M0	114	73.4 (28.1)	
M1A	1	77.6	
MX	6	82.5 (22.1)	
Histological Subtype			0.019^a^
Adenocarcinoma	96	69.4 (26.4)	
Adenosquamous	4	113.0 (30.4)	
Atypical Carcinoid	1	67.0	
Typical carcinoid	1	93.3	
Large cell	1	102.0	
Large neuroendocrine	1	102.1	
Squamous	13	88.5 (27.2)	
Small cell	1	82.4	
Other	3	77.6 (34.6)	
Differentiation			0.35
Well	12	68.8 (17.0)	
Moderate	63	72.2 (23.4)	
Poor	37	79.4 (37.0)	
Undifferentiated	1	46.5	
NA	9	72.4 (19.6)	

Features	Total	Pearson's correlation coefficient
Age (years)		0.11
Median	69	
Range	27−87	

^a^ Not significant after Dunn‐Bonferroni's.

**TABLE 3:** Healthy controls.

Features (discrete)	Total	Mean (SD) ng/mL	*p*‐value
Gender			0.21
Male	19	56.1 (10.2)	
Female	20	50.6 (16.4)	

Features	Total	Pearson's correlation coefficient
Age (year)		0.14
Median	38	
Range	21−68	

**TABLE 4:** Other non‐malignant.

Features (discrete)	Total	Mean (SD) ng/mL	*p*‐value
**Gender**			0.68
Male	11	59.3 (26.5)	
Female	15	54.0 (15.5)	
**Diseases**			0.3
Achalasia	1	20.59	
Bronchial hamartoma	1	64.98	
Chrondroid hamartoma	1	49.80	
Chronic bronchiolitis	1	21.70	
COPD	3	75.66 (12.29)	
Cyst	1	47.46	
Diaphragmatic hernia	2	60.17 (17.57)	
Esophageal mucosa with reactive changes	1	39.26	
Esophageal stromal tumour	1	50.29	
Fibroelastotic nodule	1	49.85	
Focal Atypia	1	40.62	
Granuloma	1	37.17	
Interstitial lung disease	2	88.63 (16.64)	
Pleuroparenchymal fibroelastosis	1	43.30	
Pneumothorax	1	51.49	
Pulmonary sequestration	1	46.56	
Schwannoma	1	48.77	
Sclerosing pneumocytoma	1	65.32	
Thymolipoma	2	48.88	
Zenkers diverticulum	1	119.70	
NA	1	71.77	

Features	Total	Pearson's correlation coefficient
Age (year)		
Median	65	0.5
Range	34−86	

To assess if TIMP1 could be used to differentiate tumours from healthy or benign lesions, we performed quantitative PCR (qPCR) in resected lung tumours and its surrounding healthy tissue. In agreement with previous results,^22^, ^23^ we found that TIMP1 expression was significantly higher in the tumours than in the surrounding lung parenchyma (Figure 3C). Furthermore, accurate diagnosis between preinvasive/minimally invasive cancers (non‐solid nodules) and solid nodules containing invasive lesions using CT is currently challenging.^24^ We took advantage of a recently published dataset containing RNA‐sequencing of normal lung, non‐solid nodules, and solid nodules to interrogate whether TIMP1 could be used to differentiate them and aid in clinical decisions.^24^ We found that solid nodules express significantly higher TIMP1 levels when compared to non‐solid nodules and normal lung (Figure 3D).

Additionally, IHC staining for TIMP1 in human lung tumours follows a cellular distribution similar to the tumours of the KL mice. Positive TIMP1 staining is observed in stromal fibroblasts and macrophages surrounding the tumour cells. Some high‐grade tumour cells also show positive cytoplasmic staining of TIMP1, particularly squamous cell carcinomas and adenocarcinomas with sarcomatous features (Figure 3E). This prompted us to further assess which cells from the tumour microenvironment contribute to the production of TIMP1 using a previously published single‐cell RNA‐Seq data set from human lung cancer.^18^ Although TIMP1 was detected in the epithelial/tumour cells, macrophages, myeloid‐derived suppressor cells, and monocytes; the cells with the highest expression for TIMP1 were fibroblasts (Figure S3A‐C). We further interrogated the expression of TIMP1 in lung cancer fibroblast subpopulations using a recently published single‐cell RNA‐Seq data set.^20^ We found that TIMP1 had higher relative expression in tumour adventitial and alveolar fibroblasts when compared to the same populations in healthy lungs. Interestingly, there was no difference in TIMP1 expression between tumour and healthy tissues in myofibroblasts, which are associated with poor prognosis.^20^ However myofibroblasts have the highest mean expression of TIMP1 of the three fibroblasts subpopulations, either in healthy lung or tumour (Figure S3D).

To better understand the conditions that lead to higher expression of TIMP1 in tumours, we performed a transcriptomics correlation analysis of TIMP1 using tumour RNA‐Sequencing data from the KL mice recently published by our group.^17^ We focused on the correlations of TIMP1 expression with the cytokines and chemokines that shape the tumour microenvironment. We found that TIMP1 expression correlates with proinflammatory cytokines such as tumour necrosis factor (TNF)‐α, IL‐1 α/β and IL‐6 family members such as IL‐6, IL‐11, leukemia inhibitory factor (LIF), ciliary neurotrophic factor (CNTF) and Oncostatin M (Figure S3E).

Together, these data suggest that TIMP1 is produced by the tumour and surrounding stromal cells in a pro‐inflammatory milieu, and its concentration in plasma could be used as a biomarker for lung cancer detection.

### 3.4 High TIMP1 levels correlate with poor survival in humans with lung cancer

**FIGURE 4:**
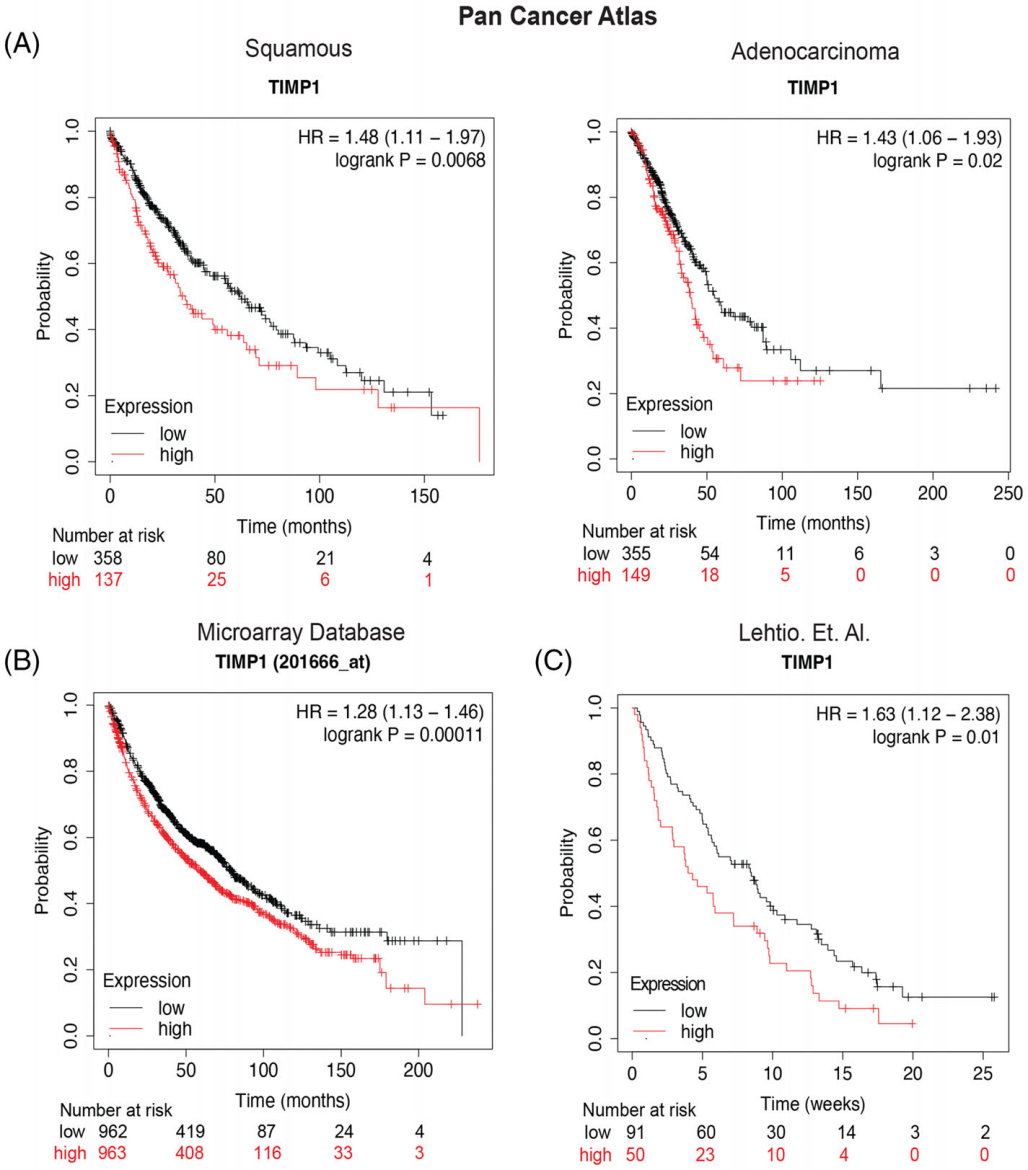
High tumour expression of TIMP1 is associated with unfavorable prognosis. (A) Kaplan–Meier (KM) survival probability graph using TIMP1 mRNA expression in squamous cell carcinoma (left) and adenocarcinoma (right) from the PanCancer Atlas. (B) KM analysis of combined microarray databases from caBIG, GEO and TCGA repositories using TIMP1 as variable factor. (C and D) KM survival probability graph using TIMP1 relative proteomic expression level in lung cancer. Comparisons in A/B/C/D were made using the Log‐rank Mantel‐Cox test (*p ≤ 0.05).

To assess the role of TIMP1 as a prognostic factor in patients with lung cancer, we mined publicly available proteomic and transcriptomic datasets (Figure 4A–C).^25^ We first assessed survival probability in the PanCancer Atlas from the TCGA consortium and found that higher TIMP1 mRNA expression is correlated with unfavorable prognosis both in Squamous‐cell and Adenocarcinoma (logrank *p* value = 0.0068 and 0.02, respectively). These results match those obtained by mining combined databases (caBIG, GEO and TCGA) of lung cancer microarrays, as depicted in Figure 4B (log‐rank *p* value: 0.00011). Furthermore, we used a recently published dataset of lung cancer that contains overall survival and proteomics of the tumours and found that higher protein levels of TIMP1 correlate with unfavorable prognosis.^26^ Overall, these data suggest that high TIMP1 mRNA and protein levels in lung tumours are associated with reduced survival probability.

## 4 DISCUSSION

Currently, there are no clinically useful serological biomarkers for lung cancer detection. In this study, we used a well‐established, genetically engineered mouse model of lung cancer to identify TIMP1 as a tumour‐secreted factor that predicts poor survival in mice and humans with lung cancer. Our results agree with those of others who found that the expression of TIMP1 correlates with poor survival in many tumour types.^23^, ^27^, ^28^, ^29^, ^30^ For example, Blanco‐Prieto et al. found that TIMP1 is significantly increased in patients with lung cancer albeit with a lower predictive AUC of 0.625, possibly because of higher levels detected in their healthy controls.^31^

TIMP1 is highly expressed in a plethora of tumours including colon,^32^ pancreas,^33^ breast,^34^ gastric,^35^ esophageal,^36^, ^37^ diffuse large B‐cell lymphoma,^38^ and renal cell carcinoma,^39^ in addition to non‐neoplastic tissues like the adipose tissue.^40^, ^41^ This wide range of tissue types suggests that the source of TIMP1 might not only be the tumour cell but a shared component of the tumour microenvironment. Using single‐cell RNA‐Seq data from human primary lung cancers, we found that the highest expression of TIMP1 occurs in fibroblasts, endothelial cells, monocytes and pericytes matching the immunohistochemistry staining pattern that we found in both human and mice lung tumours. Using bulk RNA‐Seq of the KL mice, we showed that TIMP1 expression correlates with a pro‐inflammatory tumour microenvironment. In agreement, it has been previously reported that TIMP1 secretion can be induced by treating human macrophages with IL‐6.^42^ Furthermore, another member of the IL‐6 family, Oncostatin M is a potent stimulus to produce TIMP1 in primary fibroblasts.^43^ TIMP1 is also believed to foster tumour growth by inducing MAPK signalling in the tumour^44^ and activating cancer‐associated fibroblasts.^45^ This suggests that TIMP1 may play a key role in integrating inflammation with tumour growth.

The early detection of lung cancer relies on screening high risk populations using LDCT, which may lead to false positives.^2^ For example, the NLST study showed that 24% of the surgically removed nodules were benign.^2^ The addition of a serum biomarker like TIMP1 to this testing may improve the diagnostic value of LDCT and avoid unnecessary procedures. In agreement with our findings, Birse et al. included TIMP1 as a component of an 8‐marker panel to accurately identify subjects with stage I lung cancer from high‐risk smokers using a proteomic analysis.^46^ Given the relatively high expression levels of TIMP1 in both humans and mice, it may also be possible to measure it in the material retrieved from fine needle biopsies, bronchioalveolar gavage (BAL), or embedded surgical resections via IHC, where it has been previously found in high levels in lung cancer patients.^47^, ^48^ Furthermore, TIMP1 concentration in these materials could provide additional prognostic information as higher TIMP1 mRNA and protein levels were associated with poor survival in publicly available databases.

Our study has a few notable limitations. First, our clinical samples consisted only of subjects with NSCLC and we did not evaluate the relationship of TIMP1 to other lung cancer types. However, Jumper et al. could not identify any significant difference in TIMP1 levels between NSCLC and SCLC.^49^ Furthermore, we were underpowered to distinguish if TIMP1 expression is associated with any oncogene in our cohort of patients. However, TIMP1 mRNA and relative protein levels in the LUAD CPTAC cohort were the same for all driver mutations (Figure S2E).^16^ While we were able to establish an association in mice between TIMP1 serum and tumour levels with tumour burden, we failed to do so in our cohort of patients correlating serum TIMP1 levels with tumour size measured by computed tomography (CT, data not shown). Although TIMP1 protein and mRNA levels in the tumour correlate with unfavorable prognosis in publicly available databases, we were not able to confirm the prognostic value of TIMP1 using locally‐derived plasma samples due to deficits in the collection of follow‐up data. However, previous studies have suggested that high circulating levels of TIMP1 are associated with unfavorable prognosis in lung cancer.^48^, ^50^ Despite these limitations, we showed that TIMP1 is a sensitive biomarker for lung cancer in mice and humans and we believe that the data presented in this manuscript could have broad implications for early diagnosis, follow up, and stratification of lung cancer patients.

## CONFLICT OF INTEREST STATEMENT

M.D.G. reports personal fees from Novartis, Pfizer and Scorpion Therapeutics for work unrelated to this topic. M.D.G. is co‐founder and shareholder of Faeth Therapeutics. N.K.A. has equity in Angiocrine Bioscience. O.E. is supported by Janssen, J&J, Astra‐Zeneca, Volastra and Eli Lilly research grants. He is a scientific advisor and an equity holder in Freenome, Owkin, Volastra Therapeutics and One Three Biotech and a paid scientific advisor to Champions Oncology. T.E.M. receives research funding from Janssen and from Pfizer, Inc. B.M.S. is a consultant for Pfizer, AstraZeneca and Flame Biosciences. B.M.S. is on the advisory board or a speaker for Pfizer, AstraZeneca, BMS and Genentech and is a Board member of the Lung Cancer Research Foundation and the AATS Foundation Council. B.M.S. discloses holding stock or salary in Pfizer, PPD (Pharmaceutical Product Development). B.M.S. is a consultant at Flame Biosciences and Galvanize Therapeutics. B.M.S.’s wife is an employee of and has financial interest in Xalud Therapeutics. All other authors declare no competing interests.

## FUNDING INFORMATION

The Lung Cancer Research Foundation, Grant Number: NIH K08 CA230318; The 2020 AACR‐ AstraZeneca Lung Cancer Research Fellowship, Grant Number: 20‐40‐12‐ DANT; The Starr Cancer Consortium; Weill Cornell Medicine.

## Supporting information

Supporting informaton

Supporting informaton

Supporting informaton

Supporting informaton

## Data Availability

Bulk RNA‐seq of the tumours of the KL mice has already been published^17^ and can be accessed in the GEO Database (accession number GSE165856). Single‐cell RNA‐seq of primary lung tumours was published by Laughney et al. and can be accessed in the GEO Database (GSE123904).^18^ Single‐cell RNA‐seq of fibroblasts in lung cancer was previously published and is accessible at https://zenodo.org/record/7400873#.ZGO1RuyZP0o.^20^
