# Supplementary figures and images for: TIMP1 is an early biomarker for detection and prognosis of lung cancer
Source: Clin Transl Med. 2023 Sep 27;13(10):e1391. doi: 10.1002/ctm2.1391 (PMC10533479; doi:10.1002/ctm2.1391)

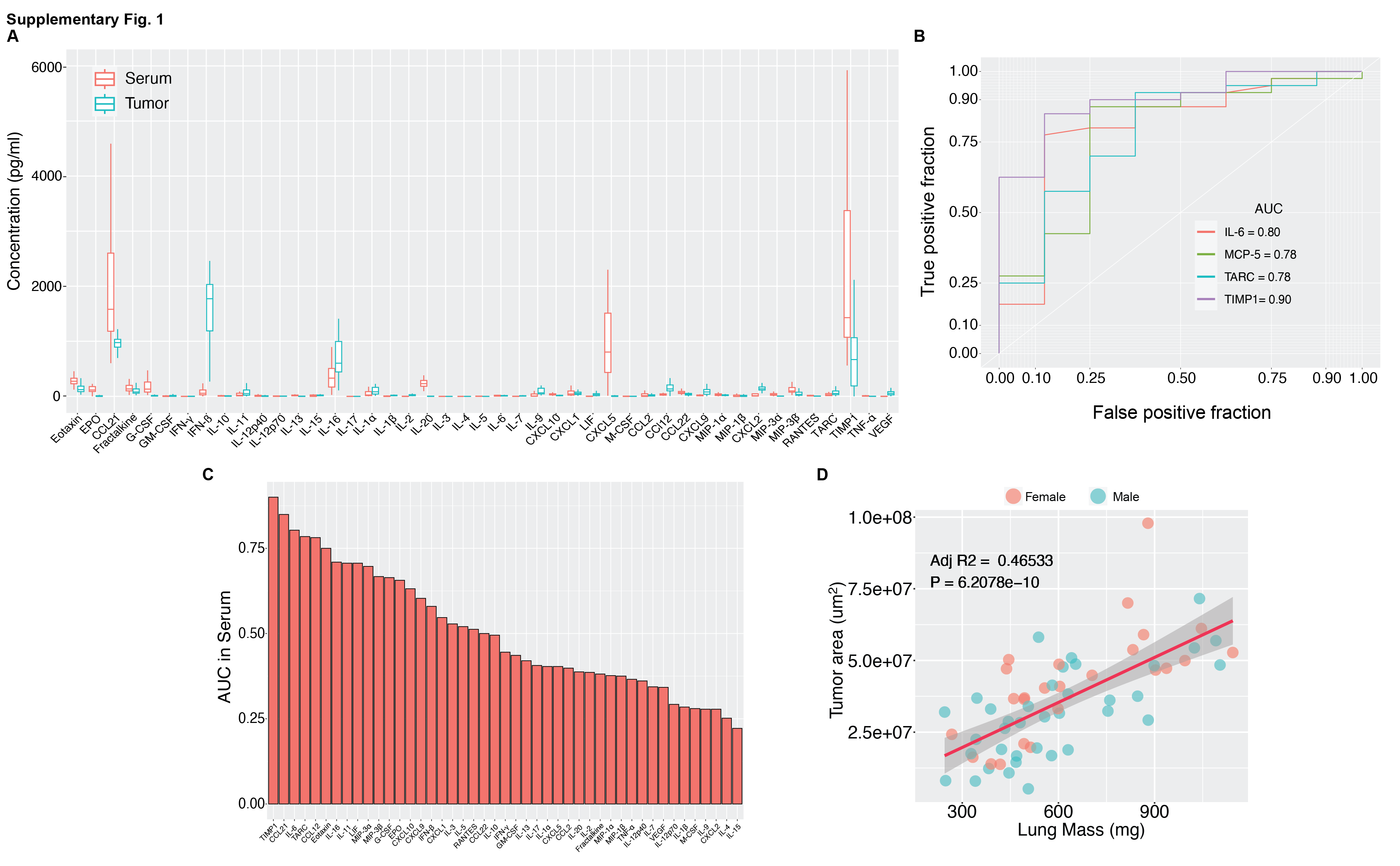

Supplement: Supplementary file 1 — Supporting informaton [file CTM2-13-e1391-s004.tif]

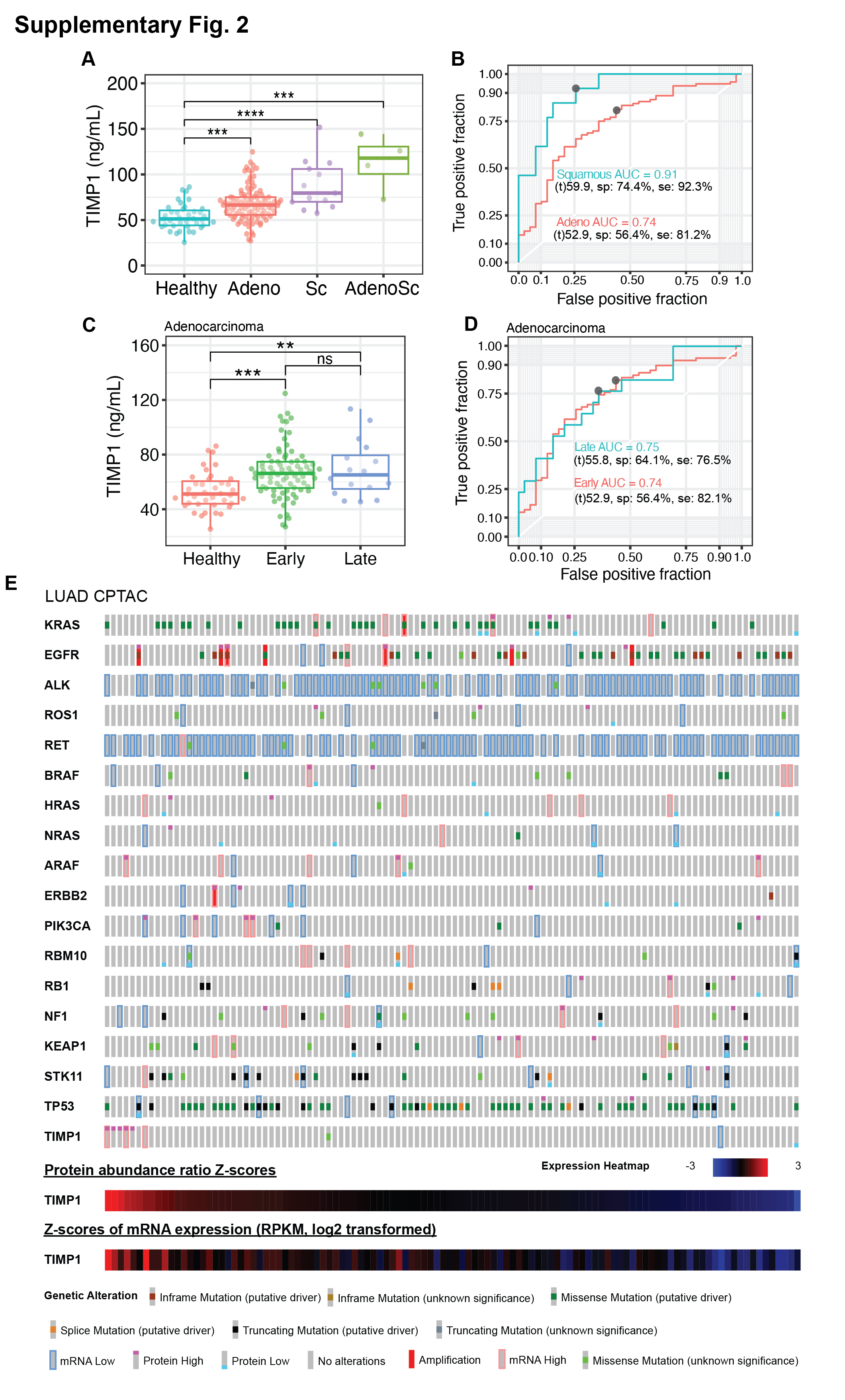

Supplement: Supplementary file 2 — Supporting informaton [file CTM2-13-e1391-s001.tif]
